# Directional Variance Adjustment: Bias Reduction in Covariance Matrices Based on Factor Analysis with an Application to Portfolio Optimization

**DOI:** 10.1371/journal.pone.0067503

**Published:** 2013-07-03

**Authors:** Daniel Bartz, Kerr Hatrick, Christian W. Hesse, Klaus-Robert Müller, Steven Lemm

**Affiliations:** 1 Machine Learning Group, Computer Science Department, Technical University of Berlin, Berlin, Germany; 2 Barclays Capital, London, Great Britain; 3 Global Markets Equity, Deutsche Bank AG, London, Great Britain; 4 Machine Learning Group, Computer Science Dept., Technical University of Berlin, Berlin, Germany; 5 Department of Brain and Cognitive Engineering, Korea University, Seoul, Korea; 6 Data Intelligence Team, Zalando GmbH, Berlin, Germany; University of Warwick, United Kingdom

## Abstract

Robust and reliable covariance estimates play a decisive role in financial and many other applications. An important class of estimators is based on factor models. Here, we show by extensive Monte Carlo simulations that covariance matrices derived from the statistical Factor Analysis model exhibit a systematic error, which is similar to the well-known systematic error of the spectrum of the sample covariance matrix. Moreover, we introduce the *Directional Variance Adjustment (DVA)* algorithm, which diminishes the systematic error. In a thorough empirical study for the US, European, and Hong Kong stock market we show that our proposed method leads to improved portfolio allocation.

## Introduction

The advent of modern finance began with Markowitz and his seminal paper on portfolio optimization [1]. His theory provides a mathematical approach to diversification by directly minimizing the portfolio variance. Moreover, by adding constraints to the optimization problem, one can e. g. prohibit or allow short-selling. Other applications comprise the creation of portfolios which constitute optimal hedges or track indices. However, a fundamental issue in portfolio allocation is the accurate and precise estimation of the covariance matrix of asset returns from historical data.

Covariance estimation and coping with its uncertainties have occupied both researchers and practitioners since then. One of the major difficulties with robust covariance matrix estimation arises from nonstationarity of financial time series [2]–[4]. Here, changes in the data generating processes force the estimation to rely on short time windows of recent observations. In addition, the number of parameters increases quadratically with the number of assets, i.e., for a set of 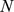 assets, the covariance matrix has 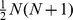 free parameters. For example, in order to estimate the covariance matrix from the daily return series of a moderately sized universe of one hundred assets, already 5050 free parameters have to be estimated. Following a general rule of thumb, that 10 observations per parameter are required for a reliable estimate, the observation window would need to cover approximately two years of data. Such a temporal horizon, however, clearly contradicts with reported nonstationarity of financial time series. In practice, the situation is even exacerbated by non-Gaussianity of financial time series [2], [5], [6], which increases the difficulty of covariance estimation even further, especially in case of small sample sizes. A possible remedy for problems caused by non-Gaussianity are robust estimation techniques [7].

As the terms *high dimensional* and *small sample size* are rather vague and interdependent, the difficulty of the task of covariance estimation is commonly characterized by the ratio of sample size to dimensionality, 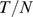, which governs the properties of the spectrum of the sample covariance matrix [8], [9]. For situations where this ratio is close to one or even below, many estimators which give better results than the sample covariance matrix have been proposed. Here, an important class is formed by regularized estimators, in which the effective degrees of freedom are reduced by Shrinkage [10]–[13]. Another way to reduce the degrees of freedom is to impose a latent structure on the data. Here, commonly factor models are in use. Factor models assume the data to be generated as a mixture of a small number of factors with additive noise [14], [15].

In this paper, we will analyse a purely statistical factor model called (Maximum Likelihood) Factor Analysis [16]. As there is no analytic solution for the parameters of the Factor Analysis model, we cannot provide a stringent theoretic analysis of its properties. Instead, by means of thorough simulations, we will provide evidence that the spectrum of the covariance matrix derived from a Factor Analysis model is biased. (We follow the terminology in [11], who deals with the bias in the spectrum of the sample covariance matrix. We do not distinguish between bias and systematic error.) To reduce the bias, we will propose the *Directional Variance Adjustment (DVA)* algorithm, which estimates the magnitude of the imposed bias in specific directions by means of a Monte Carlo sampling approach and hence enables for its correction.

In the portfolio optimization literature Monte Carlo sampling is known from Resampling Efficiency [17]. There, the authors follow a fundamentally different approach. While we use resampling to reduce the bias of the factor model, in Resampling Efficiency the sample mean and covariance are used to generate additional data sets, on which optimal portfolio weights are calculated which are then averaged. This is supposed to lead to more stable and diversified portfolios, but there is an ongoing debate on the merits of this procedure [18]. Though not based on Monte Carlo resampling, techniques for the correction of variance inflation in principal components analysis are more related to our algorithm [19], [20].

At this point we would like to emphasize that in this paper we will solely focus on the structure of risk in the stock market. A discussion of the structure of expected returns (see, e. g. 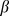-pricing models, [21]) is not within the scope of the paper.

We will evaluate our novel covariance estimation procedure in the context of portfolio optimization, where we will compare the proposed DVA Factor Analysis model to the sample covariance, Resampling Efficiency, Shrinkage, standard Factor Analysis and the Fama-French Three-Factor model [22]. By means of analyzing daily return data from 2001–2009 of three different stock markets (US, EU and Hong Kong), we will show that our proposed covariance matrix estimation scheme leads to an improved portfolio allocation and hence provide evidence that it better reflects the market’s risk structure.

The paper is organized as follows. We first review covariance estimation methods and Maximum Likelihood Factor Analysis. We will then investigate the bias in Factor Analysis by means of simulated data. Then, we will introduce our novel DVA approach for dealing with the systematic error in the model and show the effectiveness in additional simulations. Finally we will present the results of a thorough comparative study of various covariance estimation methods in the context of portfolio optimization.

## Methods

### Sample Covariance Matrix and Systematic Error in its Spectrum

The sample covariance matrix,
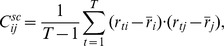
where 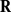 is the (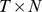)-matrix containing 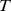 observations of 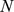 variables, is a consistent estimator of the covariance matrix: for 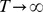 the sample covariance matrix converges to the true covariance matrix. When the ratio 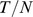 is not large, however, the sample covariance matrix tends to be ill-conditioned, implying that its inverse incurs large errors. In the extreme case, when the number of observations falls below the number of variables, the covariance matrix gets singular.

**Figure 1:**
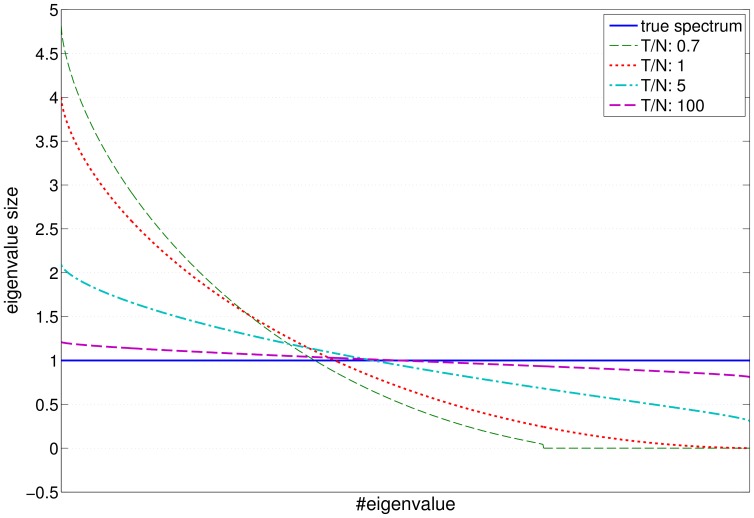
Systematic error in the spectrum of the sample covariance matrix for different ratios of sample size to dimensionality.

Though the sample covariance is an unbiased estimator of the true covariance matrix, this estimator exhibits a systematic misestimation of the spectrum of the covariance matrix which depends on the ratio of observations to dimensionality 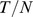. In particular, large and small eigenvalues are systematically over- and underestimated, respectively (see, e. g. [11]). In order to illustrate this systematic error, we generated empirical spectra from the Marčenko-Pastur density of eigenvalues for i.i.d. standard normally distributed variables [8]. The Marčenko-Pastur density is the eigenvalue density in the limit 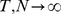, but already for sample sizes as small as 20 or 30 the empirical distribution is very similar [23]. Figure 1 shows the analytical solution for the empirical spectra for various ratios of sample sizes to dimensionality. The magnitude of the systematic error scales with the inverse of this ratio, for the degenerate case (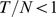) there are 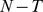 zero eigenvalues. Even for 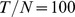, the spectrum still differs visibly from the true one.

In the literature, several methods have been proposed for correcting the spectrum. In Shrinkage [12], [13], [24], the goal is to find a suitable convex combination of the sample covariance matrix 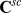 and a shrinkage target 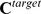,
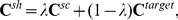
(1)where the shrinkage target is either fixed (e. g. 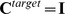) or a biased estimator with lower variance (e. g. all correlations set to their average value). For selecting the optimal shrinkage strength 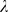, Ledoit and Wolf proposed an analytic solution [24], which is computationally faster than the commonly used model selection via crossvalidation. Shrinkage can be combined with factor modelling by taking a factor model as the shrinkage target [12]. Recently, direct shrinkage of the inverse has been proposed [25].

Random Matrix Theory (RMT, for an overview see [9]) allows for several alternative approaches to correct the spectrum. Rosenow et al. propose to retain only those eigenvalues of the correlation matrix which are larger than the largest eigenvalues of a random matrix, given by the Mar
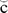
enko-Pastur law, and therefore likely to reflect some real structure [26]. The model itself is equivalent to a principipal components analysis (PCA) factor model based on the correlation matrix, where RMT is used for selection of the appropriate number of factors. Laloux et al. propose a similar model: instead of setting the eigenvalues in the bulk of the spectrum to zero, they are set to their average value [27]. A detailed analysis of these methods is beyond the scope of this article. Note that these methods are closely related to the Factor Analysis factor model -which we will discuss in the following- and thus exhibit a similiar performance and suffer from the same bias.

An interesting approach is described by el Karoui: he inverts the Marčenko-Pastur law -which describes the distribution of the sample eigenvalues- numerically in order to obtain an estimate of the true spectrum from the sample [28]. Here, one has to be aware of two facts: first, the inversion is not unique and therefore a prior or parametric ansatz has to be applied. Second, the largest eigenvalue of the covariance matrix of asset returns is normally isolated from the bulk. This is problematic, because the inversion leads to a continous spectrum. These aspects make the application of this approach less straightforward and, to our knowledge, no publication with portfolio simulations exists in which a competetive performance was achieved.

The following section introduces factor models as a type of restricted covariance estimator.

### Factor Models as Restricted Covariance Estimators

In finance, factor models form an important class of restricted covariance estimators. In a factor model, the returns 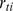 of the 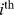 asset at time 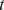 are described as a weighted sum of 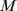 random factor returns 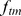 multiplied with exposures 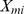 to these factors and additional random noise 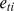:
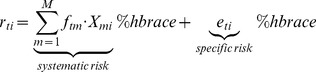
(2)

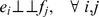


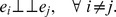



Here, the systematic risk entirely describes the dependencies between the assets, while the asset specific risks are assumed to be independent.

**Figure 2:**
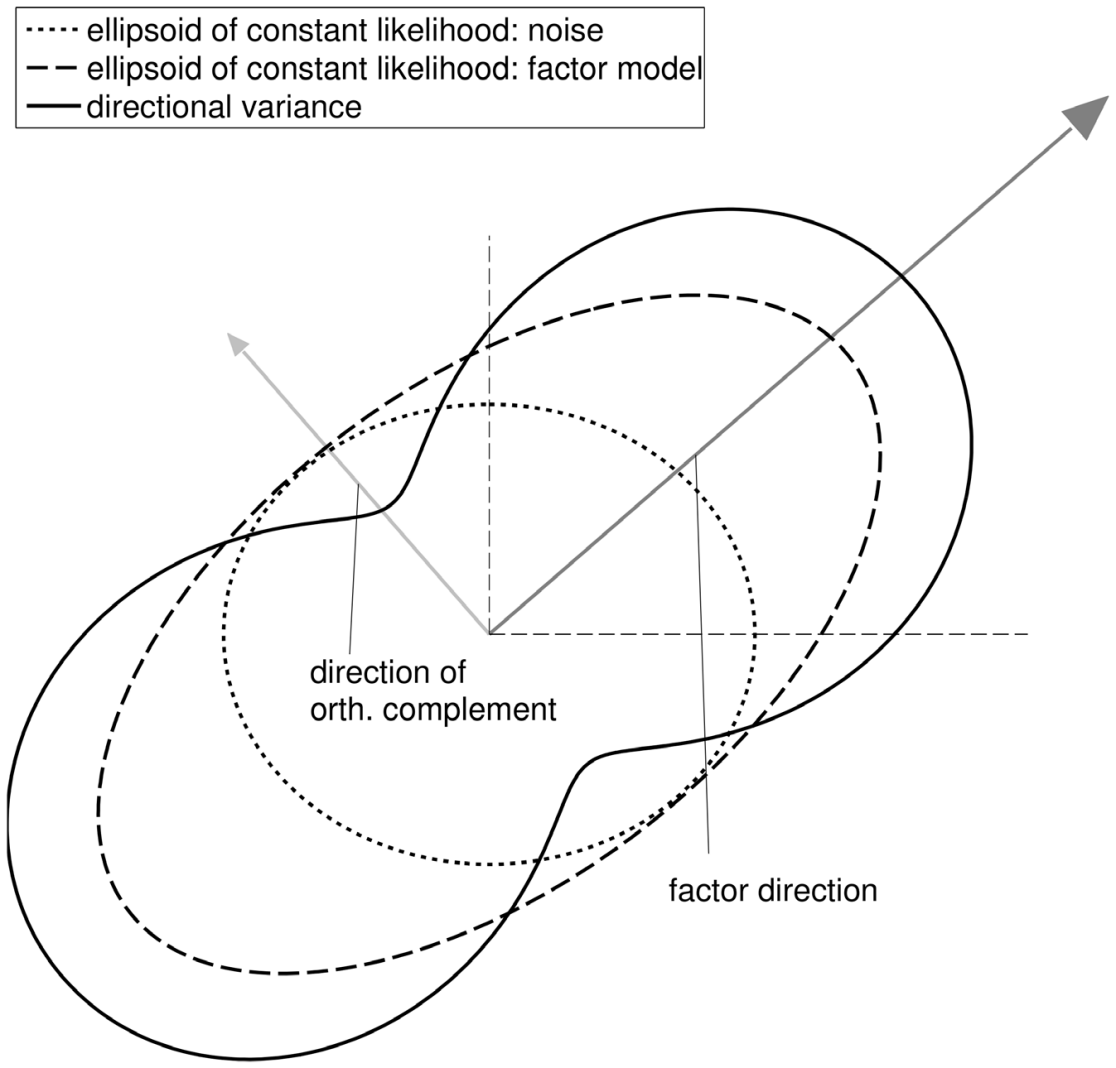
Two dimensional example of a 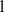-factor model. The arrows show the direction of the single factor and the orthogonal complement. The covariance matrices of the factor model 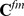 (dashed) and the uncorrelated noise 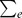 (dotted) are shown as ellipsoids of constant likelihood. The peanut-shaped solid line shows the directional variances (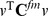) of the factor model along all directions 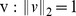.

In the statistics and signal processing literature, this is often referred to as a mixture model, where 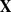 is the mixture matrix and 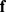 are the source signals (see, e. g., [29]). Calculating the covariance matrix, one obtains
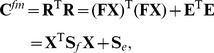
(3)where 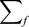 is the covariance of the factors and the diagonal matrix 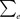 is formed by the asset specific noise variances (cf. Figure 2).

The advantage of factor models lies in the reduced number of parameters for covariance estimation. Essentially, this means that a higher bias is accepted in exchange for a reduced variance. In quantitative finance, three different types of factors are employed to build up factor models: fundamental, macroeconomic and statistical factors [4], [30].

In a fundamental factor model, assets are analysed and certain key metrics are used for setting up the factor model. Fundamental factor models are especially well suited when only a short history of data is available, e. g. for weekly or monthly data, as fewer parameters have to be estimated from the history than in a statistical factor model. The best-known model of this kind is the Fama-French three-factor model [22], in which the factor time series 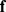 are based on portfolios governed by market beta, book-to-market ratio and market capitalization. The exposures to these factors are obtained from the coefficients of a linear regression model.

In contrast, macroeconomic factor models predetermine the factors as macroeconomic time series which are supposed to affect the asset returns. As in the Fama-French model, the exposures are obtained by linear regression. Examples for macroeconomic time series used in factor models are unemployment rate, GNP, FX or interest rates. However, for daily or higher frequency stock market returns, macroeconomic factor models are of limited use and therefore neglected in the following (for an overview, see [4]).

The third approach, statistical factor modelling, is purely data driven and extracts the factors as well as the exposures from historical asset time series. Representatives of statistical factor models are Principal Component Analysis (PCA, [31]), Probabilistic Principal Component Analysis (PPCA, [32]), Independent Component Analysis (ICA, [29], [33]), Kernel Principal Component Analysis (KPCA, [34]) as well as Factor Analysis (FA, see next section).

Hybridization combines statistical, fundamental and/or macroeconomic factors [4], [30], [35]. As long as the hybrid models contain statistical factors, our approach could be adapted to improve covariance estimation.

### (Maximum Likelihood) Factor Analysis

Factor Analysis is a latent variable model which has its roots in psychology and answers the question for the “best” explanation of the observed data for a given number of factors (latent variables). Here, “best” model refers to the model that maximizes the data likelihood. The application of Factor Analysis to financial data was first introduced in order to test the Arbitrage Pricing Theory [36].

Factor Analysis models the asset returns as a mixture of unobserved source signals with additive noise. The signals and the noise are assumed to be i.i.d., zero-mean normally distributed. Independence of the noise (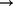 diagonal noise covariance matrix) and independence of noise and factors (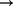 covariance is a sum of factor and noise contributions) are assumed (cf. eq. (2)). In addition, it is assumed that scaling and correlation of the systematic risk are contained in the mixing matrix (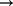 standard normally distributed independent factors). Hence, the model reads as
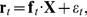
(4)

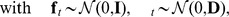
where 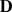 is a diagonal matrix. The corresponding log-likelihood is obtained as



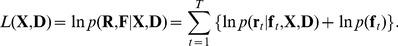
(5)Especially in the finance context, normality is a strong assumption. In order to make the model more appropriate for financial data, it is possible to extend FA to t-distributions (t-FA, [37]). t-FA has the same kind of bias as standard FA and our method can be adapted in a straightforward way by replacing FA by t-FA, but a comparison of these methods is beyond the scope of this paper.

We obtain estimates of the model parameters by Expectation-Maximization (EM, see [38], for applications on Factor Analysis see [39], [40]). In this algorithm, the likelihood is maximized iteratively by alternating between the Expectation and the Maximization step:

- in the Expectation step, the exposures 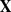 and noise variances 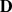 are assumed to be fixed and the expected factors 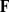 (latent variables) can be derived directly.
- in the Maximization step, the expected factors 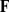 are assumed to be fixed and the likelihood is maximized with respect to exposures 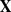 and noise variances 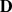.

These two steps are iterated until convergence. The resulting covariance matrix estimate of the Factor Analysis model is then given as
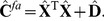
(6)


Note that the above equation follows trivially from eq. (3) for independent and standard normal factors. For Factor Analysis the number of parameters is reduced from 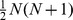 to
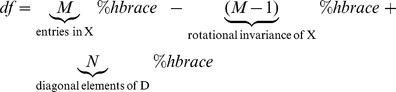


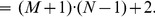
(7)


Alternative approaches to solving the optimization problem are proposed in the literature, the best known is the quasi-newton method by Jöreskog [41]. As that algorithm uses an eigendecomposition, which is costly to obtain in high dimensions (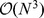), we have opted for the EM approach (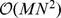). Other methods claiming superior performance suffer from the same drawback (see, e.g., [42]). Moreover, for the main claim of this paper, the optimization procedure chosen to obtain the maximimum likelihood solution is of no importance.

### Systematic Error in Factor Analysis

Unlike for the sample covariance, there are no analytical results for the spectrum of the Factor Analysis covariance matrix. Therefore, we run a simulation to study systematic errors in Factor Analysis. To this end, we generate 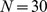 dimensional return data according to an underlying three factor model as in eq. (4). The noise covariance matrix 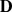 was defined with equally spaced values from the interval 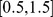 on the diagonal. The three rows of the mixing matrix 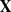 were generated as randomly oriented vectors with a length of 10, 3 and 1, respectively. In order to study the small sample size properties of Factor Analysis for this setting, we set the ratio 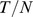 to 0.7, 1 and 5, corresponding to 21, 30, and 150 thirty-dimensional observations. As 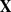 and 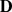 are known for the simulation, the true covariance matrix 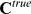 can be calculated by the population counterpart of eq. (6).

In Figure 1 we studied the systematic error of the eigenspectrum of the sample covariance matrix, where the variance in the 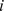-th eigendirection 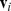 corresponds to the size of the 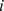-th eigenvalue 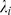:
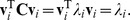



In the following we will study systematic errors in terms of misspecification of directional variances. More precisely, we will investigate systematic errors in the factor subspace and its complementary orthogonal space separately. To this end we first calculate an orthonormal basis 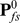 (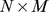) of the 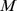-dimensional subspace in which the estimated factors 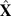 lie (the *Factor Subspace*) and another orthonormal basis 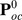 (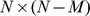) of the 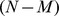-dimensional orthogonal complement. Correspondingly, we can confine the covariance matrix to the two subspaces, yielding a factor space related part and its orthogonal counterpart as
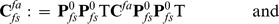


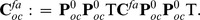



For each subspace, we obtain a new basis (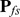 and 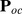) as the corresponding eigenbasis of 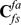 and 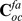, respectively. Combining these subspace bases to 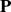 = [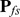,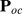] yields an orthonormal basis of the entire space in which we assue the eigenvectors sorted in decreasing order with respect to the eigenvalues.

Along these directions 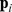 we measure the directional variances 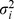 for the true and the estimated Factor Analysis model and calculate the systematic error as
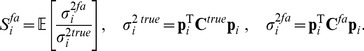
(8)


Here, values 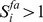 and 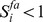 correspond to an over- and underestimation of the directional variances, respectively. Moreover, the basis 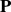 explicitly takes the factor structure into account. Hence, this particularly chosen basis enables us to study the specific systematic estimation errors in the factor subspace and noise subspace separately. Note that the directions 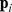 are solely derived from the estimated parameters of the factor model and do not rely on information about the true covariance matrix.

**Figure 3:**
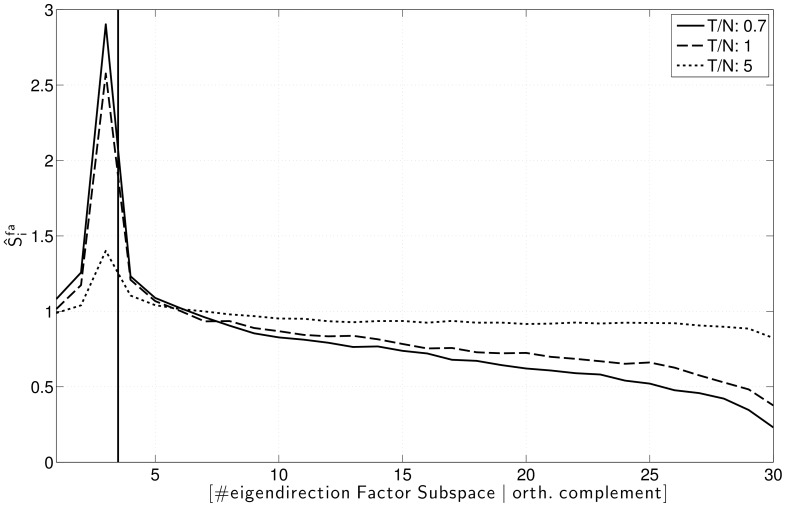
Average ratio between Factor Analysis and true variances in the factor subspace and the orthogonal complement. Ratios of sample size to dimensionality 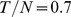, 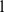 and 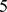. 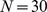. Average over 150 datasets.

Figure 3 depicts the estimated systematic error 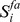 of Factor Analysis as defined in eq. (8) by means of the simulated data. Clearly, Factor Analysis tends to overestimate the variance in the 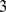-dimensional Factor Subspace, while the variance in the orthogonal complement is on average underestimated. This is not surprising, as the Factor Analysis model attributes strong covariances in the sample to the factors. Consequently, factors with low Signal-to-Noise-ratio (SNR) are hard to identify and directions of spurious covariance are likely to be misrepresented as factors, yielding an overestimating of the variance along these directions: In the simulations, the strongest (first) factor, which has a high Signal-to-Noise-Ratio can be estimated with very high accuracy even for small sample sizes and the variance estimate does not have a significant systematic error. The weaker factors with a lower SNR in contrast tend to yield overestimated variances along the estimated factor directions. This effect is highly pronounced for small sample sizes and persists for relatively large sample sizes.

On the other hand, the noise subspace spectrum shows a similar -albeit weaker- behaviour as the spectrum of the sample covariance matrix, i.e., variances corresponding to large eigenvalues are overestimated, while variances corresponding to small eigenvalues are underestimated (compare Figure 1 and Figure 3). As for the sample covariance matrix, this effect is especially pronounced for small sample sizes.

### Directional Variance Adjustment: Correcting the Systematic Error

The systematic error of the spectrum of a sample covariance matrix with respect to the true spectrum can be estimated analytically: from the distribution of the entries in the covariance matrix, one can derive the distribution of the eigenvalues (see, e.g., [9]). The minimization of the Factor Analysis cost function on the other hand does not have a closed form solution, an iterative method has to be used. Hence it does not facilitate an analytical approach to obtain the distribution of the eigenvalues. Consequently, we will deploy a method that is based on Monte-Carlo-sampling.

**Figure 4:**
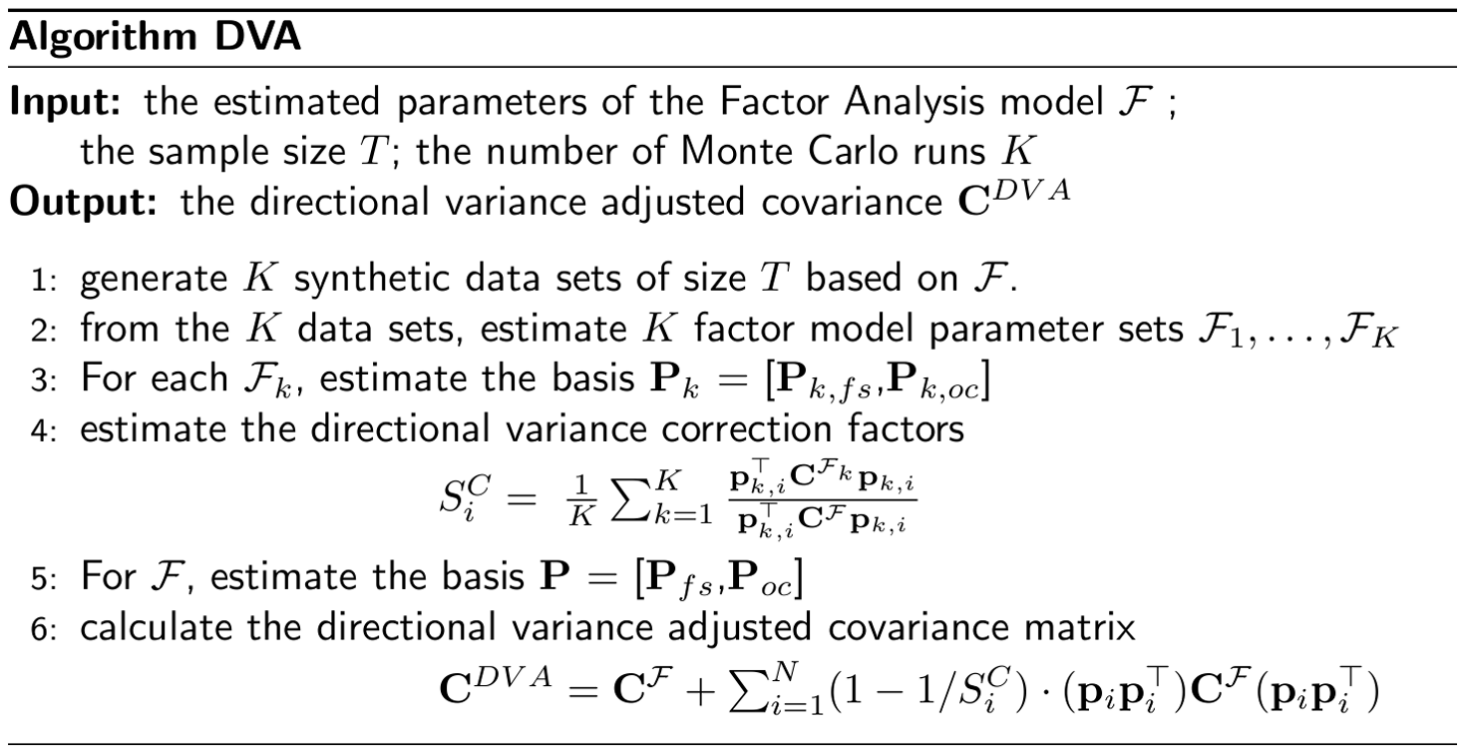
DVA algorithm.

To this end, suppose we have estimated the parameters 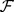 of a Factor Analysis model and want to correct the corresponding covariance matrix 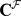 for the systematic error. Then we estimate the systematic error in the following manner: using 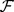 for a generative model, we generate 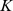 synthetic data sets of the same size as the original sample. For each data set we estimate a corresponding Factor Analysis parameter set 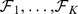. Note that for these parameter sets the true set of parameters (i.e., 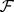) is known and with it the true covariance matrix. This enables us to quantify the amount by which the directional variances along the eigendirections of 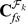 (factor subspace) and 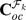 (orthogonal complement) are over- and underestimated, respectively. The estimated systematic errors, can then directly be turned into multiplicative correction factors for the adjustment of the directional variances of 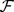. Applying these corrections to the eigendirections of the factor space and its orthogonal complement yields to what we refer as the *directional variance adjusted covariance matrix* 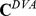 of 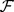 (see Figure 4).

**Figure 5:**
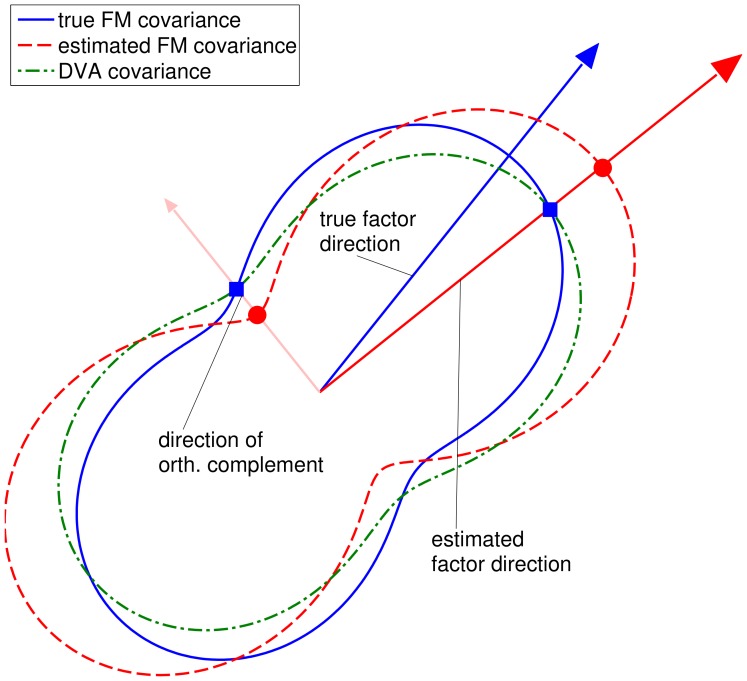
Illustration of the DVA algorithm. Depicted are directional variances for the estimated (red/dashed) and true Factor model covariance matrix (blue/solid). The blue squares indicate true variances along the estimated factor direction and the direction of the orthogonal complement. The DVA method (green/dash-dotted) aims at stretching and compressing the estimated covariance peanut such that the variances in these directions correspond to the true ones.

Note that the algorithm does not correct the parameters of the factor model itself. Instead, only the resulting covariance matrix is adjusted. In particular, the factor directions, i.e., the exposures, are kept unchanged. Figure 5 illustrates the adjusted covariance matrix. The figure shows in blue/solid and red/dashed the covariances of the true and the estimated factor model, respectively. The arrows indicate the factor directions of the true and estimated factor model and the direction of the orthogonal complement, respectively. Clearly, the factor direction has been misestimated and its strength is overestimated. In the orthogonal direction the variance is underestimated. Our proposed DVA method corrects the systematic error of the directional variance along those directions, without adjusting the directions themselves. This leads to the directional variance adjusted covariance matrix (depicted in green/dash-dotted): In the aforementioned directions, the systematic error is reduced.

One has to keep in mind that the resampling - and with it the estimate of the systematic error of the covariance matrix - is based on the estimated parameters 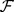. Therefore, large errors in 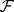 adversely affect the DVA covariance estimate.

In order to reduce the impact of the error in 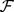, it could be advantageous to iterate the DVA procedure. From the DVA covariance matrix, which more closely reflects the true covariance matrix, we could estimate the parameters of a new factor model and restart the DVA procedure, obtaining more precise estimates of correction factors in each iteration. Though a compelling idea, there is no guarantee that iterating the DVA method will give a better solution, converge to a sensible one or even converge at all. In this paper, we therefore concentrate on the non-iterated DVA procedure.

### Simulation Results

Before we present results from daily return data, we will first illustrate the effectiveness of the proposed DVA method in a simulation study. For this, we generate toy data according to the scheme presented in the last sections, first apply standard Factor Analysis and then use our proposed DVA method to reduce the bias.

**Figure 6:**
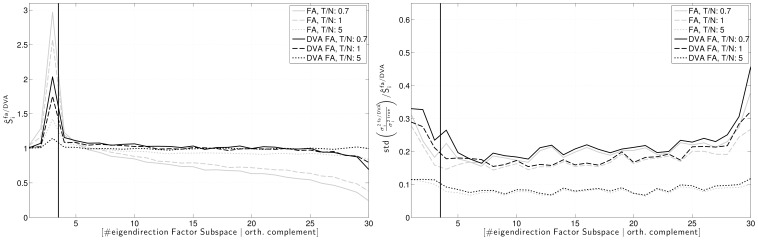
Comparison of the systematic error in standard Factor Analysis and DVA Factor Analysis. Left: systematic error. Right: normalized standard deviation of the error. Simulations for different ratios of sample size to dimensionality (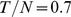, 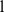 and 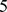). 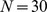. Correction factors estimated on 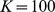 generated data sets. Mean over 150 simulations.

The performances of the two estimation methods with respect to the systematic error 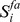 (eq. (8)) are contrasted in Figure 6. To the left, it is shown that the DVA method clearly reduces the systematic error of the Factor Analysis model, even for relatively large ratios 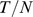. In the direction of the third factor, which has the lowest SNR, the reduction is most prominent. In the orthogonal complement of the factor subspace, the adjusted spectrum resembles the true variances very well. Nevertheless, there remains a small systematic error, which is due to to using the *estimated* parameter set in order to infer the directional variance correction factors. The right panel of Figure 6 illustrates that the DVA method does not incur a significant increase in variance of the estimate.

**Figure 7:**
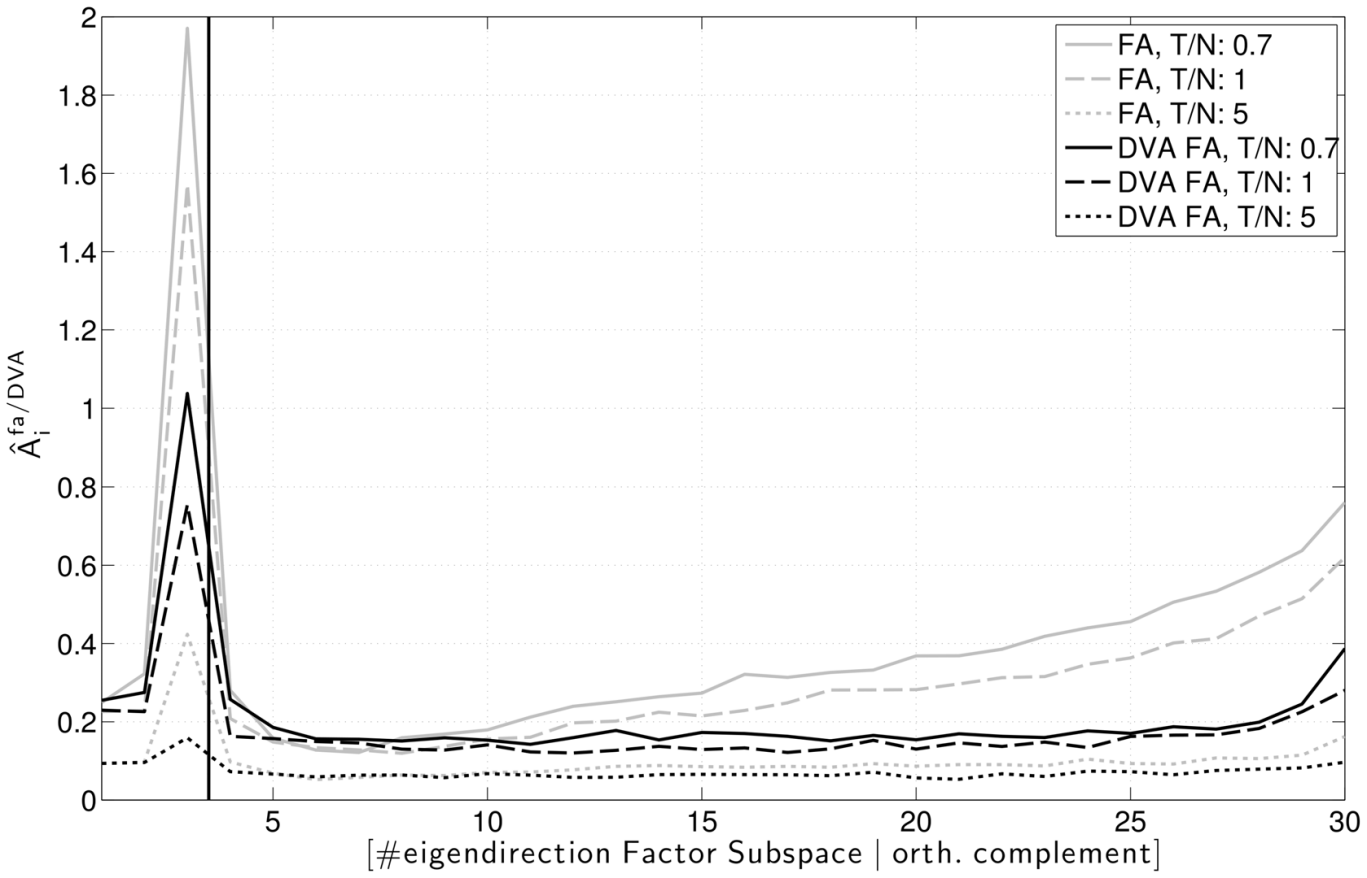
Comparison of the mean absolute relative error for standard Factor Analysis and the DVA Factor Analysis for different ratios of sample size to dimensionality (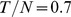, 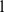 and 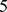). 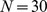
. Correction factors estimated on 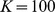 generated data sets. Mean over 150 simulations.

By reducing the systematic error without an increase in variance, the DVA method reduces the average estimation error. To account for different magnitudes of true directional variances, Figure 7 displays the error of the estimator in terms of the mean absolute relative error
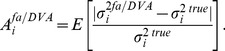
(9)


Note that this error is more than halved for the direction of the low SNR-factor and considerably decreased in the orthogonal complement. Here, DVA has the strongest effects on the directions corresponding to the largest and smallest non-zero eigenvalues of 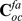. For the direction of the smallest eigenvalue, the error is again approximately halved.

**Figure 8:**
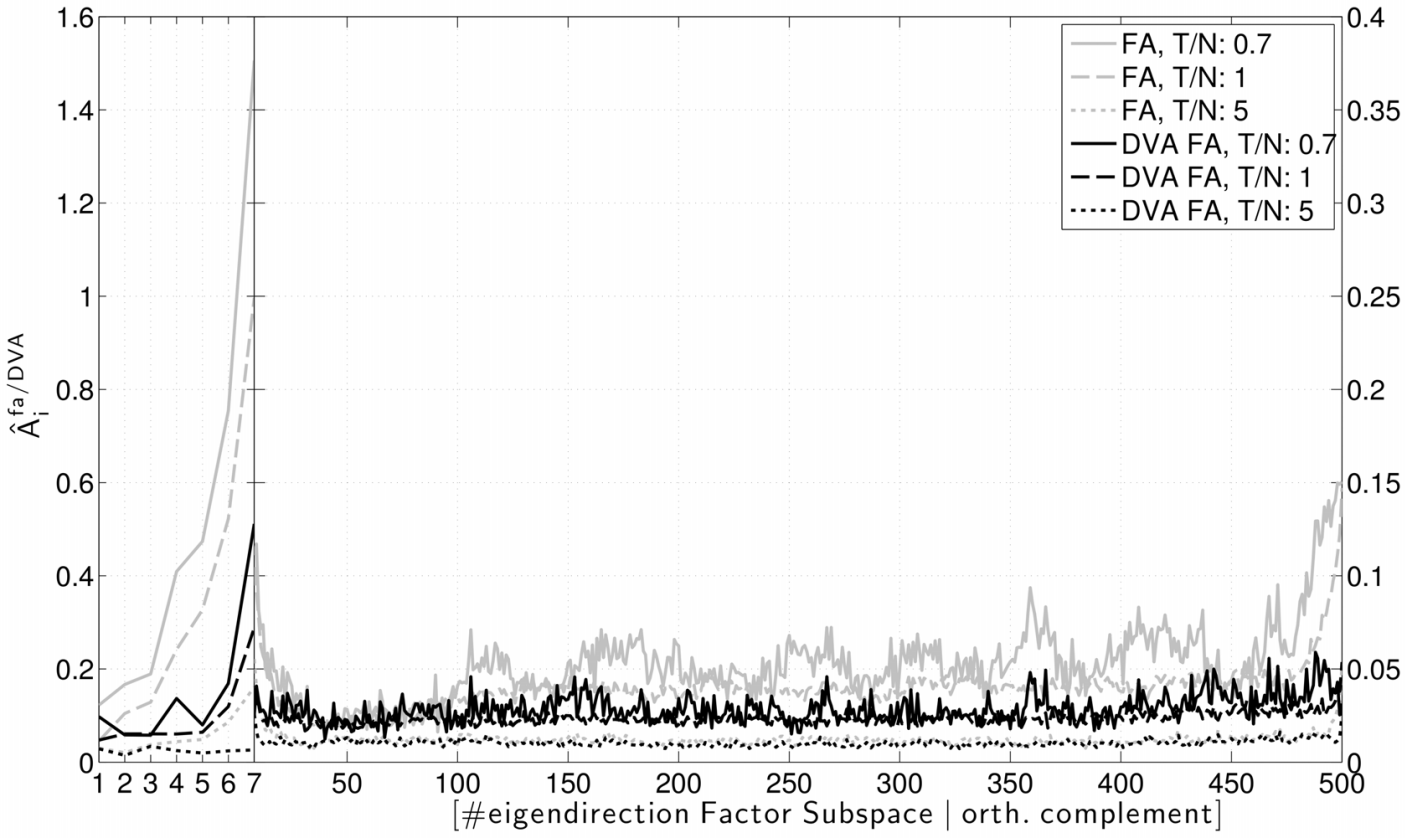
Comparison of the mean absolute relative error for standard Factor Analysis and the DVA Factor Analysis for different ratios of sample size to dimensionality (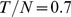, 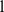 and 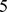). Note that the y-axis has different scaling for the factor subspace and the orthogonal complement. 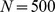. Correction factors estimated on 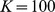 generated data sets. Mean over 150 simulations.

While the ratio 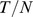 determines most properties of the sample covariance, this is not true for regularized estimators and factor models. For larger values of 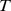, at a constant ratio 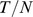, the idiosyncratic variances of Factor Analysis are estimated more precisely, while the estimation of the factors remains difficult. This is shown in Figure 8, where the dimensionality has been set to 500 and the generative model has seven factors of strength 10, 5, 4, 3, 2.5, 2, 1.5, and 1. One can see that while there is little room for improvement in the orthogonal complement, in the factor subspace the performance gain by DVA FA remains on the same level.

## Results

### Portfolio Simulation

In order to evaluate the proposed methods, we applied the DVA Factor Analysis to financial daily return time series. In the experiments, we estimate covariance matrices of stock returns and use the covariance estimates for portfolio optimization. The realized risks of the portfolios are compared for the different covariance estimates. In particular, we will compare the DVA Factor Analysis to equal weights portfolios [43], the sample covariance matrix, Resampling Efficiency (does not yield a covariance estimate) [17], the Fama-French Three-Factor model [14], [22], Ledoit-Wolf Shrinkage to a one-factor model [12] and standard Factor Analysis. For DVA and standard Factor Analysis we use seven factors. Though on the higher dimensional US and EU data sets we could extract more meaningful factors while fewer factors would be favorable on the smaller HK data set, we opted for the same intermediate model complexity on all data sets to keep the setting simpler.

### The Data Sets

The data set was aggregated from Reuters tick data. It consists of daily returns of about 1300 US stocks (3.1.2001–2.11.2009), about 600 European stocks (3.1.2001–20.4.2009) and a set of 200 stocks from the Hong Kong stock exchange (3.1.2001–26.9.2008). Removing stocks which do not have data for the whole time horizon covered by the data set, the Hong Kong data set reduces to 100 assets.

### Design of Portfolio Simulations

There are different applications of covariance matrices in portfolio optimization. Covariance matrices are needed for index tracking, hedging and the search for minimum variance portfolios. In the following, we will focus on minimum variance portfolios,
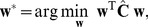
(10)where 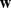 is the vector of portfolio weights and 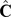 is the covariance matrix estimate.

Depending on the particular application, additional constraints are incorporated into the optimization. Commonly applied constraints include:

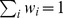
: the sum of all portfolio weights is restricted to one.

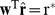
: the estimated portfolio return is restricted to 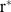, 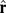 is the vector of expected/predicted asset returns.

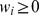
: only positive portfolio weights, no short-selling.

Note that the application of constraints tremendously prunes the set of feasible portfolios and hence diminishes the influence of the covariance estimate [44]. Consequently, the observed differences between the performances of portfolios obtained from different covariance estimation methods get smaller. Thus, in order to unveil the leverage of the various covariance estimation methods, we opted for not constraining the magnitude of the weights or enforcing their positivity. We only applied the constraint that scales the sum of the portfolio weights to one. This optimization is independent of the return estimates and is equivalent to optimizing portfolio returns under the assumption of equal expected returns for all assets.

In the case of small sample sizes, this approach will tend to overfit the directions of smallest variance and is hence expected to favour the restricted covariance estimators. Therefore, we will also investigate the performances of portfolios obtained from a regularized optimization problem of eq. (10), where the additional regularization enforces diversified portfolios.

In order to evaluate the performance of the different covariance estimator we use the realized (out-of-sample) variance of the estimated portfolios:
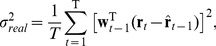
(11)and, of more financial interest, the realized mean absolute deviation



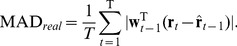
(12)In addition, we calculate the Sharpe-Ratio and the turnover. Note that (11) and (12) are rolling out-of-sample estimates, as 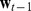 and 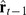 are the portfolio weights and expected returns estimated on the information available until time 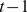. More precisely, for the estimation of the covariance matrix 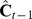 and the averaged return 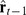 we choose a strictly causal window of 150 trading days. This size balances out two error sources: on the one hand, estimation gets more precise with additional data points. On the other hand, nonstationarity of the return distribution limits the usefulness of older data [4]. We use slightly more observations than in most studies with monthly data [12], [44], but less than e.g. Bouchaud et al. use for daily data [45]. In our experience, larger windows reduce performance.

In order to reduce the variance of the performance evaluation and to thoroughly explore the estimated covariance structure, 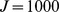 subsets, each confined to 40 (HK) or 100 (US and EU) assets, are chosen and the optimal (confined) portfolio 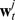 is constructed from the given covariance matrix estimate 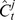. The realized variance and realized absolute deviation are then determined based on the average performance across the different confined portfolios, i.e.,
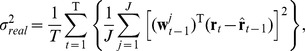


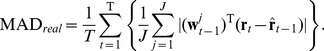



## Results and Discussion of Portfolio Simulations

In this section we will provide portfolio simulation results for different covariance estimation approaches, namely the sample covariance matrix, Resampling Efficiency, the Fama-French three-factor model, shrinkage to a one-factor Model, a Factor Analysis model with seven factors, and a directional variance adjusted Factor Analysis (DVA FA). The results for the different markets are summarized in Table 1.

**Table 1:** Portfolio risk.

	MAD	MSE	Sharpe	turnover
US				
1/N - eq. weights	10.63†	246.8†	0.61	**0.00**†
Sample Cov.	8.56†	156.1†	0.51†	6.49†
Resampling Eff.	8.83†	165.6†	0.50†	6.81†
Fama-French	5.65†	73.5†	**0.77**	2.06†
LW Shrinkage	5.56†	69.6†	0.74†	2.69†
Factor Analysis	5.47†	67.8†	0.73†	2.53†
DVA	**5.40**	**66.7**	0.72	2.33
Europe				
1/N - eq. weights	8.10†	154.8†	0.65†	**0.00**†
Sample Cov.	5.93†	78.9†	0.91	5.09†
Resampling Eff.	6.11†	83.4†	0.89	5.32†
Fama-French	3.97†	38.6†	1.16†	1.71†
LW Shrinkage	4.00†	39.1†	1.26	2.24†
Factor Analysis	3.88†	36.5†	**1.30**†	2.06†
DVA	**3.84**	**36.0**	1.29	1.91
Hong Kong				
1/N - eq. weights	10.21†	209.5†	1.17†	**0.00**†
Sample Cov.	6.57†	81.2†	1.22	2.06†
Resampling Eff.	6.64†	82.7†	1.21	2.13†
Fama-French	6.20†	73.5†	**1.37**	1.46†
LW Shrinkage	6.17†	72.9†	1.37	1.62†
Factor Analysis	6.17†	73.0†	1.37	1.64†
DVA	**6.12**	**71.7**	1.37	1.52

Mean absolute deviations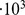, mean squared deviations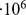, Sharpe-Ratio and turnover of the resulting portfolios for the different covariance estimators and the different markets. 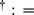 DVA mean significantly better/worse than this model at the 5% level, tested by a randomization test.

First note that the equal weights portfolio has very high risk, a result which is also reported by Kourtis et al. [25].

As expected, the sample covariance matrix is not the most suitable tool for portfolio optimization. Across all data sets, the portfolios derived from the different factor based models and Shrinkage clearly outperform the sample covariance matrix based portfolios in terms of realized risk. A direct comparison of these models reveals that the DVA method always significantly outperforms Fama-French, standard Factor Analysis and Shrinkage with respect to realized variance and realized absolute deviation. On our data sets, Resampling Efficiency does not give an advantage over the sample covariance matrix.

The Sharpe-Ratios do not give a clear picture: Fama-French, Statistical Factor Modelling and Shrinkage each perform best in one market. This is not suprising, as we did not optimize for high returns.

The turnovers on the other hand show an additional advantage of DVA Factor Analysis over standard Factor Analysis: covariance estimates are more stable.

## Results and Discussion of Portfolio Simulations - Additional Regularization

Without knowledge of the covariance structure of the assets, the best portfolio allocation would have weights inverse to the variance of the assets and hence be highly diversified. Minimization of eq. (10), on the other hand, gives the optimal portfolio only for the true covariance matrix. Therefore, for a given covariance matrix estimate, it should in principle be possible to additionally reduce the realized risk of a portfolio by increasing its diversification, e.g., by regularization of eq. (10).

Consequently, the aim of the following analysis is twofold. First of all and from a theoretical perspective, we want to investigate if the superior performance of the DVA method can be simply explained away by a higher degree of diversification or if the true covariance structure is indeed better captured. Secondly, with respect to practical considerations, we are interested in the best achievable performance.

In order to analyze these aspects, for each of the covariance matrix estimates 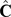 we enforce additional portfolio diversification by including a ridge penalty in the objective function eq. (10), i.e.,
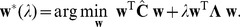
(13)


In particular, we set the metric 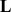 to a diagonal matrix which has the single asset variances on its diagonal. This metric implies that each asset gets penalized by its variance and in the limit 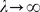 we obtain the portfolio of assets weighted by the inverse of their variances.

**Figure 9:**
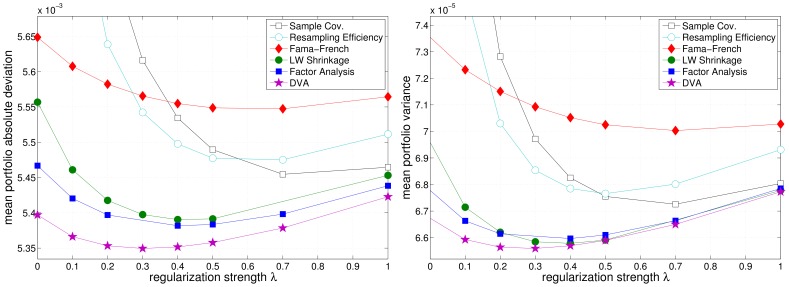
Regularization dependency of the realized portfolio risk in the US market. Left: mean absolute deviation. Right: variance.

**Figure 10:**
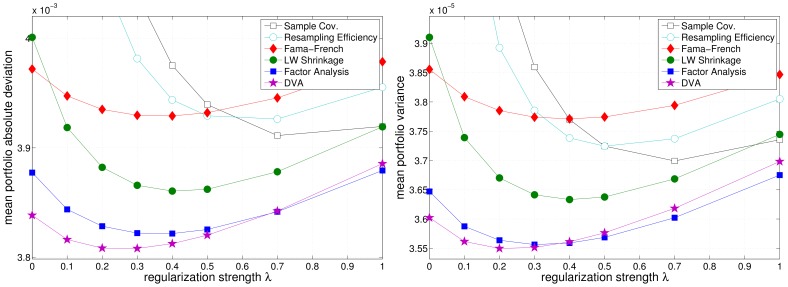
Regularization dependency of the realized portfolio risk in the EU market. Left: mean absolute deviation. Right: variance.

**Figure 11:**
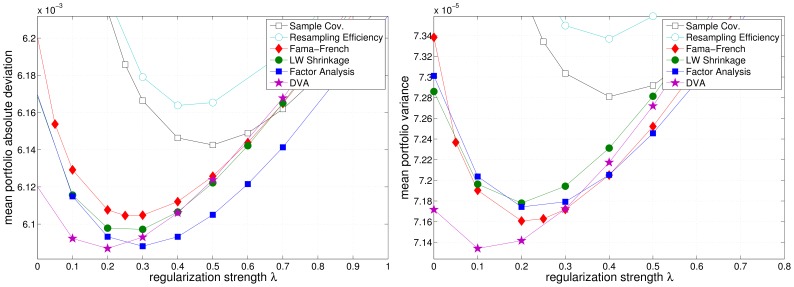
Regularization dependency of the realized portfolio risk in the HK market. Left: mean absolute deviation. Right: variance.

Figures 9, 10, and 11 depict the realized (out-of-sample) variance and MAD (see eq. (11) and eq. (12)) of the resulting portfolios as a function of the regularization parameter 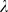 for the three different market samples. Equal weights portfolios have been omitted in the figures because they incur far higher risk. n unison, the different models benefit from additional regularization, as can be seen from a reduction of the realized risk of the resulting portfolios (cmp. Tables 1 and 2). Although, this effect is most pronounced for the sample covariance matrix, it merely reaches the performance of the (unregularized) Factor Analysis models. Note that the regularized optimization based on the sample covariance matrix is equivalent to unregularized optimization using a shrinkage covariance estimator, that employs 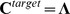 as the shrinkage target (cf. eq. (1)). Again, Resampling Efficiency does not prove to be superior to the sample covariance matrix.

**Table 2:** Portfolio risk under regularization.

	MAD	MSE	Sharpe	turnover
US				
1/N - eq. weights	9.78†	212.9†	0.61	**0.00**†
Sample Cov.	5.45†	67.3†	0.76	1.90
Resampling Eff.	5.48†	67.7	0.76	1.81
Fama-French	5.55†	70.0†	**0.77**	1.61
LW Shrinkage	5.39†	65.8	0.75	1.73
Factor Analysis	5.38†	66.0	0.74	1.75
DVA	**5.35**	**65.6**	0.72	1.65
Europe				
1/N - eq. weights	7.50†	133.6†	0.66†	**0.00**†
Sample Cov.	3.91†	37.0	1.26†	1.69
Resampling Eff.	3.93†	37.2	1.26	1.61
Fama-French	3.93†	37.7	1.17	1.44
LW Shrinkage	3.86†	36.3	1.28	1.53
Factor Analysis	3.82†	35.6	**1.30**	1.56
DVA	**3.81**	**35.5**	1.29	1.50
Hong Kong				
1/N - eq. weights	9.50†	181.7†	1.19	**0.00**†
Sample Cov.	6.14†	72.9†	1.48	1.10
Resampling Eff.	6.16†	73.4†	1.48	1.09
Fama-French	6.11	71.7	1.50	1.02
LW Shrinkage	6.10	71.8	**1.51**	1.06
Factor Analysis	6.09	71.7	1.50	1.07
DVA	**6.09**	**71.3**	1.49	1.03

Mean absolute deviations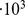, mean squared deviations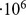, Sharpe-Ratio and turnover of the resulting portfolios for the different regularized covariance estimators for optimal regularization strength and the different markets. 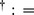 DVA mean significantly better/worse than this model at the 5% level, tested by a randomization test.

Shrinkage to the one-factor model profits as well from additional shrinkage to 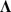. This indicates that the optimization of the expected mean squared error the analytic shrinkage formula yields a too small shrinkage parameter for the optimization of portfolios.

Surprisingly, the Fama-French Three-Factor model benefits less from regularization than Shrinkage, although the unregularized performance is similar. This implies that the performance gain of the unregularized Fama-French model over the sample covariance matrix is mainly due to a strong imposed prior towards highly diversified portfolios. Compared to the statistical factor models FA and DVA FA, the performance difference remains on the same level as without additional regularization. This means that the covariance structure is better captured by the statistical Factor models than by the Fama-French model. These effects are strongest for the US and EU markets.

The risk of the portfolios obtained from the Factor Analysis model as well as from its DVA version also improve considerably. At the optimal degree of regularization, the DVA FA model significantly outperforms the optimally regularized sample covariance matrix based model for all markets. Regarding eq. (13) as being a shrinkage towards 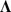, this statement is equivalent to: shrinkage of the DVA Factor Analysis covariance matrix towards 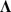 yields better portfolios with respect to the achieved portfolio risks than shrinkage of the sample covariance matrix towards 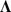. The comparison of DVA FA with Fama-French shows a significantly better performance for all markets as well. The performance gain over Shrinkage is, however, only significant for US and EU markets.

At the optimal degree of regularization the difference in performance between the standard Factor Analysis and the DVA Factor Analysis is reduced. In general, this was to be expected as regularization can equivalently be achieved either by adding a penalty term to the objective function or by additionally constraining the feasible set. In this respect, it was shown that the actual influence of the covariance matrix estimate on the minimum variance portfolio diminishes when additionally constraining the set of feasible portfolios [44]. Thus, as a matter of fact, regularization partly compensates for the influence of the systematic error of the Factor Analysis covariance matrix estimate.

Nevertheless, in the US and EU market, the peformance gain in MAD of DVA over standard Factor Analysis remains significant at the 5% level. In Hong Kong the difference is -for optimal regularization- not significant.

Comparing the different markets, it turns out that the Hong Kong market shows a slightly different behavior than the American and European. At the Hong Kong market, all methods likewise benefit from additional diversification. One possible explanation is that the HK data set contains quite a few outliers and missing data as opposed to the US and EU data. Thus covariance estimates as well as least square estimates of factor exposures are hampered in general. Hence and in contrast to the other markets, the Fama-French model also clearly profits from the additional regularization, although its overall performance remains inferior to DVA Factor Analysis.

### Conclusions

The fundamental issue in portfolio allocation is the accurate and precise estimation of the covariance matrix of asset returns from historical data. Among many challenges, the data is typically high dimensional, noisy, contaminated with outliers and nonstationarity interferes with the use of long estimation windows. Thus, reliable statistical parameter estimation is impeded. Our work has contributed to alleviate this problem in theoretical and practical aspects: we (1) demonstrated that the data driven statistical Factor Analysis model has a systematic estimation error (2) proposed the algorithmic Directional Variance Adjustment (DVA) framework, which alleviates this bias, and finally (3) provided extensive simulations of minimum variance portfolios of EU, US and Hong Kong markets, underpinning the usefulness of the DVA approach in terms of significant gains in realized variance and realized mean absolute deviation. Compared to standard Factor Analysis, covariance estimates are more stable and turnover is reduced. or each covariance estimator, we additionally studied the effect of regularizing the minimum variance portfolios towards a higher degree of diversification. As expected, diversification improved portfolio performance across the different estimators. Our empirical study showed that while regularization slightly decreases the overall advantage gained by DVA, the remaining difference in the minimum stayed significant for the US and EU data sets, here the DVA Factor Analysis method is superior to standard Factor Analysis.

A second interesting finding of the regularization experiments was that the advantage of the Fama-French model over the sample covariance matrix estimator appears rather due to an imposed strong diversification prior than to an improved estimation of the underlying covariance structure. Here, clearly the combination of regularization and statistical factor models like standard FA and in particular DVA FA led to better model performance.

Note, however, that down-weighting/regularizing away the estimated correlations may not always be a valid option. In an application where the covariance structure is of higher importance - e.g. because an index needs to be tracked with a reduced number of assets - increased diversification would clearly be no option.

Therefore, both scenarios, the one with and the one without regularization, yield interesting insight and provide clear evidence for the superiority of DVA FA.

Whilst we have studied and modeled daily returns, the DVA method is of course equally capable of being employed to derive covariances for intraday returns. Intraday covariance matrices are particularly relevant when dealing with portfolios with significant (intraday) churn. Examples of such portfolios include internalization portfolios at most major brokerages, and those used for market making. Using DVA FA, a covariance matrix may be tuned for the typical period a position remains in a portfolio, allowing, potentially, better risk management and asset allocation.

We do not consider serial correlation, as it is common for covariance estimation methods like Shrinkage [12], [24] and statistical factor models (see, e.g., [4]). Nevertheless, it would be interesting to do further research on an autoregressive Factor Analysis model.

## References

[1] Markowitz H (1952) Portfolio selection. Journal of Finance VII: 77–91.

[2] Loretan M, Phillips PC (1994) Testing the covariance stationarity of heavy-tailed time series. Journal of Empirical Finance 1: 211–248.

[3] Pagan AR, Schwert GW (1990) Testing for covariance stationarity in stock market data. Economics Letters 33: 165–170.

[4] Connor G, Goldberg L, Korajcyzk R (2010) Portfolio Risk Analysis. Princeton University Press.

[5] Longin F (2005) The choice of the distribution of asset returns: How extreme value theory can help? Journal of Banking and Finance 29: 1017–1035.

[6] Campbell RA, Forbes CS, Koedjik KG, Kofman P (2008) Increasing correlations or just fat tails? Journal of Empirical Finance 15: 287–309.

[7] Huber PJ (1981) Robust statistics. Wiley Series in Probability and Mathematical Statistics. John Wiley & Sons.

[8] Marčenko VA, Pastur LA (1967) Distribution of eigenvalues for some sets of random matrices. Mathematics of the USSR-Sbornik 1: 457.

[9] Edelman A, Rao NR (2005) Random matrix theory. Acta Numerica 14: 233–297.

[10] Stein C (1956) Inadmissibility of the usual estimator for the mean of a multivariate normal distribution. In: Proc. 3rd Berkeley Sympos. Math. Statist. Probability. volume 1, 197–206.

[11] Friedman JH (1989) Regularized discriminant analysis. Journal of the American Statistical Association 84: 165–175.

[12] Ledoit O, Wolf M (2003) Improved estimation of the covariance matrix of stock returns with an application to portfolio selection. Journal of Empirical Finance 10: 603–621.

[13] Schäfer J, Strimmer K (2005) A shrinkage approach to large-scale covariance matrix estimation and implications for functional genomics. Statistical Applications in Genetics and Molecular Biology. 10.2202/1544-6115.1175 16646851

[14] Fan J, Fan Y, Lv J (2008) High dimensional covariance matrix estimation using a factor model. Journal of Econometrics 147: 186–197.

[15] Goldfarb D, Iyengar G (2003) Robust portfolio selection problems. Mathematics of Operations Research 28: 1–38.

[16] Basilevsky A (1994) Statistical Factor Analysis and Related Methods. John Wiley & Sons, Inc.

[17] Michaud RO (1998) Efficient Asset Management: A Practical Guide to Stock Portfolio Optimization and Asset Allocation. Oxford University Press, USA.

[18] Scherer B (2004) Resampled efficiency and portfolio choice. Financial Markets and Portfolio Management 18: 382–398.

[19] Kjems U, Hansen LK, Strother SC (2001) Generalizable singular value decomposition for ill-posed datasets. Advances in Neural Information Processing Systems 13: 549–555.

[20] Abrahamsen TJ, Hansen LK (2011) A cure for variance ination in high dimensional kernel principal component analysis. J Mach Learn Res 12: 2027–2044.

[21] Shanken J (1992) On the estimation of beta pricing models. Review of Financial Studies 5: 1–34.

[22] Fama EF, French KR (1992) The cross-section of expected stock returns. Journal of Finance XLVII: 427–465.

[23] Tulino AM, Verdú S (2004) Random matrix theory and wireless communications. Commun Inf Theory 1: 1–182.

[24] Ledoit O, Wolf M (2004) A well-conditioned estimator for large-dimensional covariance matrices. Journal of Multivariate Analysis 88: 365–411.

[25] Kourtis A, Dotsis G, Markellos RN (2012) Parameter uncertainty in portfolio selection: Shrinking the inverse covariance matrix. Journal of Banking and Finance 36: 2522–2531.

[26] Rosenow B, Plerou V, Gopikrishnan P, Stanley HE (2002) Portfolio optimization and the random magnet problem. Europhys Letters : 500–506.

[27] Laloux L, Cizeau P, Potters M, Bouchaud JP (2000) Random matrix theory and financial correlations. International Journal of Theoretical and Applied Finance 3: 391–397.

[28] el Karoui N (2008) Spectrum estimation for large dimensional covariance matrices using random matrix theory. Annals of Statistics 36: 2757–2790.

[29] Hyvärinen A, Oja E (2000) Independent component analysis: algorithms and applications. Neural Netw 13: 411–430. 10946390 10.1016/s0893-6080(00)00026-5

[30] Connor G (1995) The three types of factor models: A comparison of their explanatory power. Financial Analysts Journal 51: 42–46.

[31] Jolliffe IT (1986) Principal component analysis. Springer Series in Statistics. Springer.

[32] Tipping ME, Bishop CM (1999) Probabilistic Principal Component Analysis. Journal of the Royal Statistical Society, Series B 61: 611–622.

[33] Comon P (1994) Independent component analysis, a new concept? Signal Process 36: 287–314.

[34] Schölkopf B, Smola A, Müller KR (1998) Nonlinear component analysis as a kernel eigenvalue problem. Neural computation 10: 1299–1319.

[35] Miller G (2006) Needles, haystacks and hidden factors. Journal of Portfolio Management 32: 25–32.

[36] Roll R, Ross SA (1980) An empirical investigation of the arbitrage pricing theory. Journal of Finance XXXV: 1073–1103.

[37] McLachlan G, Bean R, Jones LBT (2007) Extension of the mixture of factor analyzers model to incorporate the multivariate t-distribution. Computational Statistics and Data Analysis 51: 5327–5338.

[38] Dempster AP, Laird NM, Rubin DB (1977) Maximum likelihood from incomplete data via the EM algorithm. Royal statistical Society B 39: 1–38.

[39] Rubin D, Thayer D (1982) EM algorithms for ML factor analysis. Psychometrika 47: 69–76.

[40] Roweis S, Ghahramani Z (1999) A unifying review of linear gaussian models. Neural Computation. 10.1162/089976699300016674 9950734

[41] Jöreskog KG (1967) Some contributions to maximum likelihood factor analysis. Psychometrika 32: 443–482.

[42] Zhao JH, Yu PLH, Jiang Q (2008) ML estimation for factor analysis: EM or non-EM? Statistics and Computing : 109–123.

[43] DeMiguel V, Garlappi L, Uppal R (2009) Optimal versus naive diversification: How inefficient is the 1/N portfolio strategy? Review of Financial Studies 22: 1915–1953.

[44] Jagannathan R, Ma T (2003) Risk reduction in large portfolios: Why imposing the wrong constraints helps. Journal of Finance LVIII: 1651–1683.

[45] Bouchaud JP, Potters M (2011) Financial applications of random matrix theory: a short review. In: The Oxford Handbook of Random Matrix Theory, Oxford University press.

